# Plasticity in ascending long propriospinal and descending supraspinal pathways in chronic cervical spinal cord injured rats

**DOI:** 10.3389/fphys.2012.00330

**Published:** 2012-08-17

**Authors:** Marie-Pascale Côté, Megan R. Detloff, Rodel E. Wade, Michel A. Lemay, John D. Houlé

**Affiliations:** Department of Neurobiology and Anatomy, Drexel University College of MedicinePhiladelphia, PA, USA

**Keywords:** spinal cord injury, propriospinal, supraspinal, inter-enlargement, motor-evoked potentials, magnetic stimulation, H-reflex

## Abstract

The high clinical relevance of models of incomplete cervical spinal cord injury (SCI) creates a need to address the spontaneous neuroplasticity that underlies changes in functional activity that occur over time after SCI. There is accumulating evidence supporting long projecting propriospinal neurons as suitable targets for therapeutic intervention after SCI, but focus has remained primarily oriented toward study of descending pathways. Long ascending axons from propriospinal neurons at lower thoracic and lumbar levels that form inter-enlargement pathways are involved in forelimb-hindlimb coordination during locomotion and are capable of modulating cervical motor output. We used non-invasive magnetic stimulation to assess how a unilateral cervical (C5) spinal contusion might affect transmission in intact, long ascending propriospinal pathways, and influence spinal cord plasticity. Our results show that transmission is facilitated in this pathway on the ipsilesional side as early as 1 week post-SCI. We also probed for descending magnetic motor evoked potentials (MMEPs) and found them absent or greatly reduced on the ipsilesional side as expected. The frequency-dependent depression (FDD) of the H-reflex recorded from the forelimb triceps brachii was bilaterally decreased although H_max_/M_max_ was increased only on the ipsilesional side. Behaviorally, stepping recovered, but there were deficits in forelimb–hindlimb coordination as detected by BBB and CatWalk measures. Importantly, epicenter sparing correlated to the amplitude of the MMEPs and locomotor recovery but it was not significantly associated with the inter-enlargement or segmental H-reflex. In summary, our results indicate that complex plasticity occurs after a C5 hemicontusion injury, leading to differential changes in ascending vs. descending pathways, ipsi- vs. contralesional sides even though the lesion was unilateral as well as cervical vs. lumbar local spinal networks.

## Introduction

As the majority of human injuries are incomplete and occur at the cervical level (www.sci-info-pages.com), experimental models of cervical SCI are highly clinically relevant. Functional deficits include impaired fore- and hindlimb function due to damage to the white matter that affects both descending and ascending systems, and to the gray matter containing the segmental circuitry for processing sensory input and generating motor output.

Long ascending propriospinal axons are likely candidates to be involved in coupling and coordination between the cervical and lumbar central pattern generators (CPGs; Miller et al., 1973a,b; English et al., 1985) as the rhythmicity in cervical segments can be driven by the lumbar CPG (Juvin et al., 2005). A subgroup of long propriospinal axons ascend in the ventrolateral funiculus (VLF) and projects to cervical lateral motoneuronal pools, giving them a direct influence on forelimb control (Sterling and Kuypers, 1968; Giovanelli and Kuypers, 1969). Propriospinal neurons have recently emerged as attractive targets for treatment after spinal cord injury (SCI) as regeneration over long distances is very limited. They have the potential to form new intraspinal circuits (Bareyre et al., 2004; Courtine et al., 2008) and regenerate (Fenrich and Rose, 2009) after SCI, giving these pathways a high potential of success if integrated in a comprehensive combinatory treatment such as cellular transplant and neurotrophin delivery (Jordan and Schmidt, 2002; Conta and Stelzner, 2004; Fouad et al., 2005).

Because the lesion characteristics evolve over an extended period of time after the initial spinal injury, non-invasive non-noxious methods are warranted to address the potential effect of injury/treatments but not many electrophysiological outcome measures of this nature are available or clinically relevant. Previous studies showed that magnetic inter-enlargement responses (MIER) and magnetic motor-evoked potentials (MMEPs) are reproducible, non-invasive and represent an objective assessment of axonal conduction and functional integrity in the ascending intersegmental lateral funiculus and descending VLF, respectively (Loy et al., 2002; Beaumont et al., 2006). This study characterizes plasticity occurring in the intact ascending propriospinal pathways after an incomplete cervical injury and describes concomitant changes in the function of descending supraspinal pathways as well as cervical and lumbar spinal local circuits.

## Materials and methods

All procedures were performed in accordance with protocols approved by Drexel University College of Medicine Institutional Animal Care and Use Committee and followed National Institutes of Health guidelines for the care and use of laboratory animals.

### Surgical procedures and post-operative care

A total of 38 adult female Sprague-Dawley rats (225–250 g, Charles River) were used for this study. Table 1 summarizes the condition and testing for each group of animals. A cervical hemicontusion injury was performed on thirty-one animals. Animals were anesthetized with a mixture of ketamine (60 mg/kg), xylazine (6 mg/kg), and acepromazine (6 mg/kg) and spinal cord injuries performed as previously described (Sandrow et al., 2008; Sandrow-Feinberg et al., 2009). Briefly, a unilateral cervical laminectomy (C5) was performed and a moderate unilateral contusion injury was created by an impact force of 200 Kdyne with tissue displacement to a depth of 1600–1800 μm using the Infinite Horizon Impact Device (Precision Systems and Instrumentation, Lexington, KY). Upon completion of surgery, overlying muscles were sutured and the skin incision closed with wound clips. Animals were given dextrose in saline (5 mL, s.c.), buprenorphine (0.05 mg/kg, i.m.) for 3 days as an analgesic, and ampicillin (100 mg/kg, s.c.) for 7 days to prevent infection. Bladders were expressed manually as needed.

**Table 1:** **Number of animals in behavioral, electrophysiological, and histological section of the study**.

**Group**	***n* =**	**Behavior**	**White matter sparing**	**MMEP**	**MIER (magnetic)**	**MIER (electric)**	**H-reflex**
Hemicontusion	11	X	X	X	X		
	15	X	X	X	X		X
	5^*^				X	X	
Intact	5	X	X				X
	2	X			X	X	

* This group had a survival time of 2 weeks.

### Behavioral assessments

After acclimation to the testing apparatus over a 2 week period, baseline scores were established for each animal. Due to the unilateral nature of our injury model, we evaluated the ipsilesional (right) and contralesional (left) limbs separately. Right and left hindlimb and forelimb scores were recorded preoperatively and weekly thereafter for 6 weeks.

#### Open field locomotion

Forelimb and hindlimb function was evaluated using the Forelimb Locomotor Scale (FLS; Sandrow et al., 2008) and the Basso, Beattie, Bresnahan Locomotor Rating Scale (BBB; Basso et al., 1995), respectively. The FLS is an 18-point scale that ranks ipsilesional forelimb locomotion after unilateral cervical SCI based on range of motion, degree of weight support, and paw placement. The FLS scale is not sensitive enough to identify compensation on the contralesional forelimb, thus, only data for the ipsilesional forelimb is included. The BBB is a 22-point scale which ranks hindlimb locomotion after SCI from complete paralysis (0) to normal and coordinated movement (21).

#### CatWalk locomotion

Interlimb coordination was assessed preoperatively and at 6 weeks after SCI on the CatWalk using methods described previously by Kloos et al. (2005). Six passes of runway locomotion for each testing session were processed and analyzed for footfall patterns. Three aspects of coordination were quantified: the patterns of limb placement, the consistency by which the rat was able to implement this pattern and the ratio of limb placements by calculating the mean phase dispersion, the mean standard deviation of the phase dispersion, and the incidence of mismatches, respectively, across trials for diagonal (Ipsilesional Forelimb-Contralesional Hindlimb (IF-CH; CF-IH), girdle (CF-IF; CH-IH), and ipsilateral (IF-IH; CF-CH) pairings. To quantify the pattern of interlimb coordination, we measured the synchrony or phase dispersion of initial contact between different limbs. Phase dispersion is defined as the degree of synchrony between two limbs during locomotion. For limb pairings that are “in phase” (i.e., RF-LH or LF-RH), initial contact of stance occurs at the same time for the limb pairing and yield a phase dispersion of 0%. For limb pairs that alternate (i.e., ipsilateral RF-RH or girdle LF-RF), typically yield a phase dispersion of 50% and appear out of phase, as one limb of the pairing is in stance while the other limb is in swing. Importantly, when the initial contact of one limb follows the diagonal limb, the phase dispersion value is expressed as positive. Conversely, when the initial contact of one limb precedes that of the diagonal limb, the phase dispersion value is reported as negative. During locomotion of SCI animals, the degree of synchrony between limb pairings is compromised. When the phase dispersion or timing of limb pairings drifts beyond 75%, a mismatch occurs. The number of mismatches of diagonal pairs is summed for each pass and the percentage of mismatches to the total number of step cycles was determined.

### Electrophysiological assessments using magnetic stimulation: inter-enlargement responses and motor-evoked potentials

**Figure 1:**
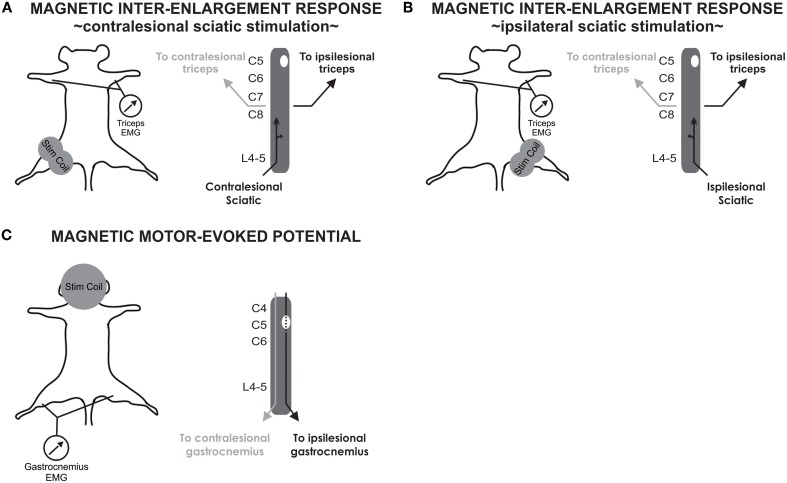
**Experimental procedure for stimulation and recordings of MIER and MMEP. (A)** MIERs are evoked with a figure of eight coil positioned at the hip to stimulate the contralesional sciatic nerve. Recordings are performed in the triceps brachii bilaterally. **(B)** MIER recordings were additionally performed with the coil positioned on the ipsilesional hip. **(C)** MMEPs are evoked with a circular coil positioned over the cranium while recording in the gastrocnemius muscle bilaterally.

Magnetic inter-enlargement responses (MIERs) were measured to assess conductivity in propriospinal ascending pathways between the lumbar and cervical enlargement (Beaumont et al., 2006; Cao et al., 2010). Magnetic motor-evoked potentials (MMEPs) were measured to evaluate impairment of transmission in descending motor pathways using methods described elsewhere (Magnuson et al., 1999; Loy et al., 2002). Magnetic stimulation was performed in awake, non-sedated animals positioned on a wooden board. Animals were restrained using a piece of stockinet pinned to the board around the animal. The four limbs remained accessible for electrode placement. EMGs were recorded via 27-gauge stainless steel needle electrodes (Cadwell Laboratories, Kennewick, WA) inserted into either the forelimb triceps brachii (MIER) or hindlimb gastrocnemius muscle (MMEPs). The active electrode was placed in the muscle belly whereas the reference electrode was placed near the distal tendon of the muscle. The ground electrode was placed subcutaneously in the neck area (MIER) or at the base of the tail (MMEP). Magnetic stimulation was administered using a Magstim 200^2^ stimulator (The Magstim Company, Wales, UK) delivering monophasic stimuli (100 μs approximate rise time, 1 ms duration). Figure 1 illustrates the stimulus and recording configuration. MIERs were elicited via a double 25 mm coil (figure of eight) positioned at the hip to stimulate the sciatic nerve. MMEPs were evoked by transcranial magnetic stimulation delivered via a 50 mm circular coil held over the rat cranium to elicit EMGs in distal musculature. The 25 mm coil generates 4.6 T magnetic field whereas the circular 50 mm coil generates 3.6 T (www.magstim.com). The stimulation intensity is expressed as the percent of absolute maximal output of the stimulator. In each case, stimulation was elicited with a series of increasing intensities (40, 60, 80, 100%). To determine the motor threshold (MT), the stimulus intensity was decreased by intervals until no response was evoked. The MT was defined as the stimulation intensity at which a response was evoked at least 50% of the time. Each assessment included the determination of the MT followed by recordings at 60 and 80%. A minimum of five responses were recorded at each of two intensity levels and averaged.

To confirm the MIER results, electrical stimulation of the sciatic was performed in five contused animals 2 weeks after SCI and also in three normal animals. This time point was chosen because our results showed that MIER responses were already modulated at 1 week post-SCI. A cuff electrode was surgically implanted on the sciatic nerve and the animal recovered from isoflurane anesthesia. Recordings similar to those described above were performed in the awake animal “suspended” on a vinyl cloth platform with the hindlimbs hanging free beneath the animal. Single bipolar pulses of 500 μs total duration were applied with intensities of 1 mA.

EMG signal was amplified (×100–1000, A-M Systems), bandpass filtered (10–300 to 5000 Hz), digitized and fed to a custom software (Labview, National Instruments). Averages were computed and peak-to-peak amplitude and onset latency (initial deflection from baseline) were measured for each animal, at each time point and intensity of stimulation.

### H-reflex recordings and analysis

Eight weeks after SCI, a terminal experiment was performed to assess the frequency-dependent depression (FDD) of the H-reflex in all 4 limbs of 14 of the contusion injured animals and 5 normal animals. Rats were anaesthetized with a mixture of ketamine (60 mg/kg), xylazine (10 mg/kg) and acepromazine (6 mg/kg). H-reflex was recorded as previously reported for the hindlimbs (Côté et al., 2011) and forelimbs (Hosoido et al., 2009). Briefly, the tibial (hindlimbs) or ulnar nerve (forelimbs) was isolated, dissected free and mounted on a bipolar hook electrode for stimulation. Skin flaps were used to form a pool of mineral oil to prevent dessication of the nerves throughout the recording period. EMG was recorded using bipolar wire electrodes (Cooner Wire, Chatsworth, CA) inserted in the hindlimb or forelimb interosseus muscles. H-reflexes were evoked via an isolated pulse stimulator (A-M Systems, Carlsborg, WA) delivering single bipolar pulses (100 μs each phase) to the tibial or ulnar nerve. Stimuli of increasing intensities were used to determine the MT, the H-reflex threshold and to determine the maximal response amplitude for both M and H-wave (M_max_ and H_max_). The stimulation intensity which elicited H_max_ response (~1.2 MT) was then used for 3 series of 17 consecutive stimulation pulses delivered at 0.3 Hz, 5 Hz or 10 Hz. The 0.3 Hz series was then replicated. The trial was discarded if the M-wave amplitude was not within 95% of the initial 0.3 Hz control series. EMG recordings were amplified (×1000, A-M Systems) and bandpass filtered (10-5k Hz). Signal was digitized (10 kHz) and fed to custom software.

Response latency (onset of response) and peak-to-peak amplitude were measured for the H and M responses evoked by single pulses. The recruitment curve was plotted by expressing the amplitude of the H and M responses as a function of stimulus intensity. The H_max_/M_max_ ratio, which is believed to give an estimate of motoneuronal excitability, was calculated to assess the relative proportion of motoneurons recruited through the monosynaptic reflex loop as compared to the activation of the entire motor pool. The H_max_/M ratio was also calculated to estimate the relative activation of the motor pool required to reach maximal reflex amplitude. For the analysis of FDD, the first five responses to a train of stimulation were discarded to allow reflex stabilization and the last twelve responses were averaged for every stimulation frequency (0.3, 5, and 10 Hz). H-reflex amplitude was normalized to M_max_ and the change in H-reflex response at 5 Hz and 10 Hz was calculated as a percentage of the response measured at 0.3 Hz (control).

### Lesion analysis

Rats were given an overdose of Euthasol (390 mg/kg sodium pentobarbital, 50 mg/kg phenytoin, ip.) and perfused transcardially with 4% paraformaldehyde. Cervical spinal cord between C4 and C6 was removed, post-fixed in paraformaldehyde at 4°C overnight and submersed in 30% sucrose for cryoprotection. Transverse serial sections throughout the rostral to caudal extent of the lesion 250 μm apart were mounted on glass slides, air-dried and stained for cresyl violet (Sigma) for Nissl and euriochrome cyanine (Sigma) for myelin. Sections were cover slipped with Vectashield mounting medium (Vector Laboratories, Burlingame, CA).

To determine the amount of spared tissue, the contralesional gray and white matter of the spinal cord and spared gray and white matter on the ipsilesional side were measured separately in 10 sections (250 mm apart) spanning the rostrocaudal extent of the lesion using the Cavalieri estimator method (Stereo Investigator, MicroBrightfield, Burlington, VT). To quantify the degree of spared tissue at the lesion epicenter, we figured the proportion of the spared tissue area on the ipsilesional hemisphere to the spared tissue area on the contralesional (uninjured) spinal cord (Sandrow-Feinberg et al., 2009). To further delineate regions within the white matter that are thought to be responsible for eliciting MMEP or MIER responses (Magnuson et al., 1999; Loy et al., 2002), we used a pie-shaped overlay that had twelve equal wedges. The center of the overlay was aligned with the central canal (see schematic in Figure 6. To determine the effects of sparing on MIER, the proportion of spared tissue for wedges 3 and 4 was calculated and the data were partitioned into groups based on the amplitude of their MIER (high amplitude >9.7 mV or low amplitude. The effect of sparing on MMEP was determined by calculating the proportion of spared tissue for wedge 5 only. For these analyses, animals were grouped based on the presence or absence of an ipsilesional MMEP. Correlational analysis determined whether a relationship exists between the amplitude of the MIER or MMEP response to the proportion of spared tissue in these specified regions of the lesion epicenter.

### Statistical analysis

One-Way ANOVA followed by Holm–Sidak *post-hoc* test were used to determine significant differences across groups for all data unless stated below. If the sample variables did not fit a normal distribution or were not equally variant, a One-Way ANOVA on ranks followed by Dunn's *post-hoc* test was performed. All data are reported as mean ± SEM. Statistical analysis was performed using Sigma Plot software 11.0 and PASW Statistics 18. For all statistical tests, the significance level was set to *p* < 0.05. A Two-Way ANOVA followed by Holm–Sidak *post-hoc* test was used to assess whether stimulation frequency and treatment group had a significant effect on the amplitude of the H-reflex and to evaluate if the interaction of these factors affected the variable. Correlation analysis using Spearman's Rank Correlation was used to determine a relationship between the amplitude of the MMEP or MIER responses to spared tissue at the lesion epicenter.

## Results

### Anatomical and behavioral measures

**Figure 2:**
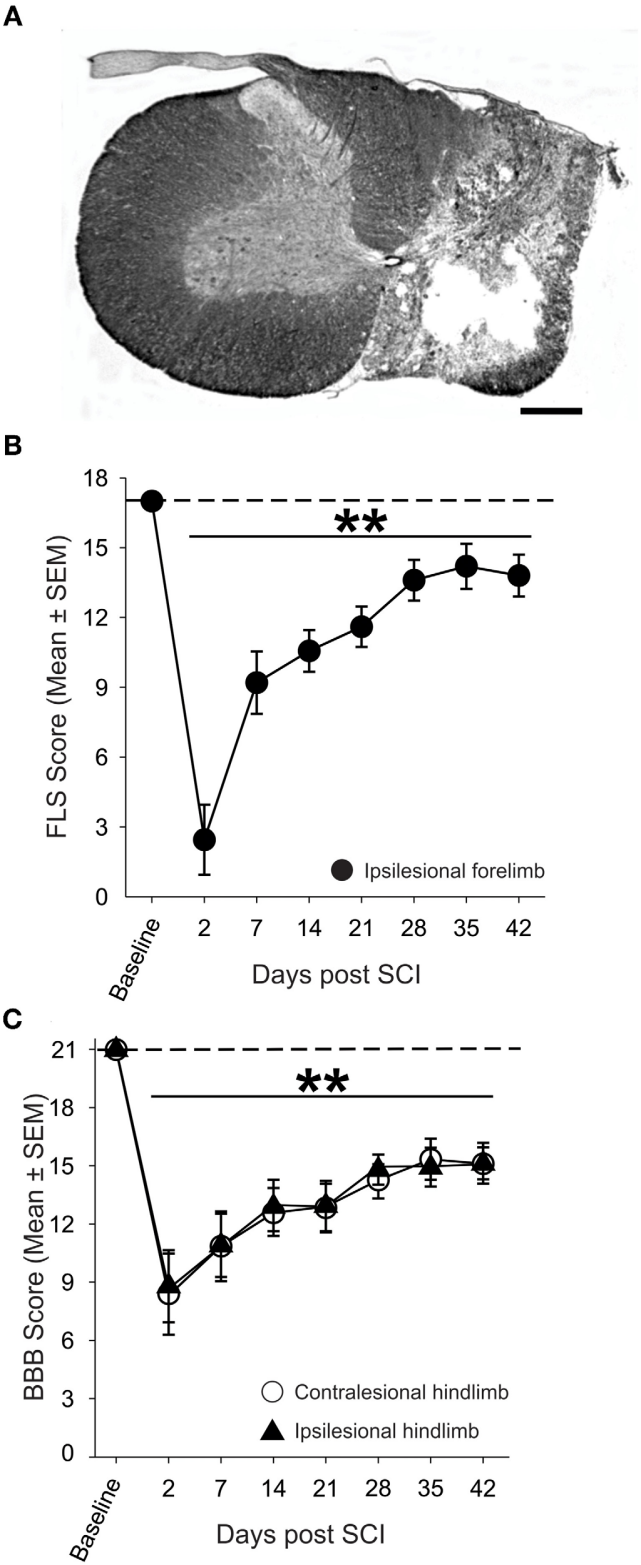
**Anatomical and behavioral evaluation of unilateral spinal cord contusion. (A)** Representative transverse section of the lesion epicenter stained for Nissl and myelin. 200 k dyne impact produced a unilateral lesion with a spared rim of lateral white matter on the right side of the spinal cord, while the gray and white matter of the contralesional spinal cord appears normal (scale bar = 0.5 mm). **(B)** Assessment of the ipsilesional forelimb with the Forelimb Locomotor Scale (FLS) revealed significant deficits in ipsilesional forelimb function acutely after SCI that partially recovers over time. **(C)** Unilateral SCI produced significant, bilateral deficits in hindlimb locomotor function in the open field as assessed by the Basso, Beattie, Bresnahan (BBB) Locomotor Rating Scale.

Nissl-myelin staining revealed that contusive SCI produced a core lesion limited to one side of the spinal cord (mean lesion area of 1.52 ± 0.09 mm^2^) that nearly eliminated all gray matter and spared a thin rim of myelinated tissue in the lateral and ventral funiculi (1.09 ± 0.10 mm^2^; Figure 2A). Contiguous myelin staining and nuclear labeling in the contralesional spinal cord indicated that the ascending and descending spinal cord tracts and gray matter remain normal (3.44 ± 0.13 mm^2^). On average less than one-third of the ipsilesional spinal cord was spared at the lesion epicenter of contused rats.

The ability of SCI animals to utilize their ipsilesional forelimb in the open field was assessed using the FLS (Figure 2B). Two days after SCI, animals exhibited extensive movement of one forelimb joint or slight movement of two forelimb joints (FLS score <3; *p* < 0.01 vs. baseline). By four weeks after SCI, animals exhibited continuous plantar stepping of the ipsilesional forepaw with parallel paw position and occasional toe clearance (FLS score =14; *p* < 0.01 vs. baseline). Assessment of the hindlimbs using the BBB scale revealed that unilateral cervical contusion caused bilateral deficits in hindlimb locomotion that persisted for the duration of the study (Figure 2C). At one week after SCI, all animals were able to frequently or consistently plantar step with both hindlimbs, but they were unable to coordinate forelimb and hindlimb movements (BBB score = 11) at this early post-injury interval. Spontaneous locomotor recovery reached a plateau by four weeks after SCI but deficits in trunk stability, paw placement and toe clearance persisted in all animals. Importantly, just over 80% of animals demonstrated consistent FL-HL coordination at 4 weeks after injury.

### Transmission in ascending inter-enlargement pathways is enhanced on the ipsilesional side after incomplete SCI

**Figure 3:**
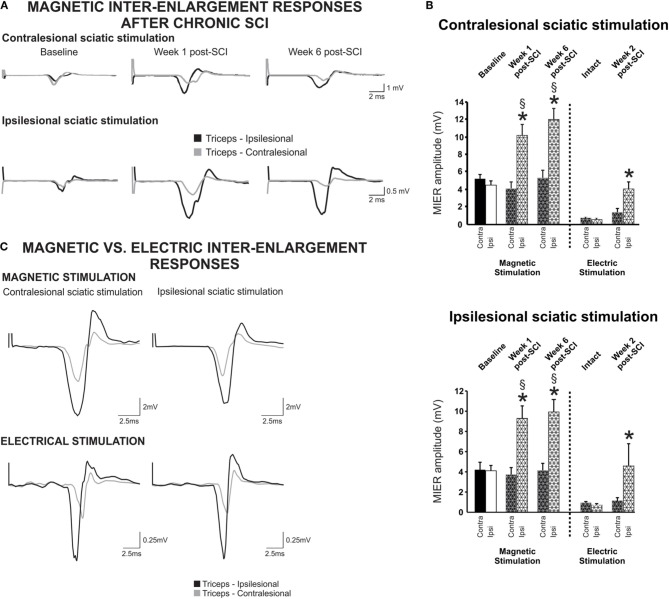
**Inter-enlargement responses are enhanced on the ipsilateral side of the lesion after a cervical hemicontusion injury. (A)** Inter-enlargement responses are recorded in the ipsilesional and contralesional triceps muscles following magnetic stimulation of the ipsilesional or contralesional sciatic. Representative traces recorded from a single animal illustrate that the response in the ipsilesional triceps (black) is larger than in the contralesional triceps (gray) after SCI. The MIER is also delayed, i.e., the latency in the contralesional triceps is significantly longer than in the ispilesional side whether the stimulation is evoked from the ispilesional or contralesional sciatic. **(B)** Regardless of ipsi- or contralesional stimulation, the amplitude of the magnetically and electrically-evoked inter-enlargement response in the ipsilesional triceps is significantly increased as compared to baseline and as compared to the contralesional triceps. **(C)** Inter-enlargement responses recorded from the same animal with magnetic (top panel) or electrical (bottom panel) stimulation of the contralesional or ipsilesional sciatic nerve. Note that the magnetic and electric stimulation elicit similar responses in the ipsilesional and contralesional triceps. EMG responses are larger in the ipsilesional triceps than in the contralesional triceps. In addition, the response in the contralesional triceps is delayed as compared to baseline or ispilesional triceps.

Conductivity and connectivity of ascending spared/reorganized fibers after cervical hemicontusion injury were estimated using MIER. The MIER was evoked by stimulating magnetically the ipsi- or contralesional sciatic nerve and recording from the triceps brachii bilaterally in awake animals. We found out that maximal response was reached at 60% of the maximum output of the stimulation unit. To avoid possible occlusion of the response, all results below were obtained at 60% of the stimulator output. Figure 3A displays representative recordings of MIER at baseline, 1 and 6 weeks after contusion injury. The latency, the time it takes for the magnetic impulse to travel from the stimulation site to the recording electrode, i.e., from the stimulus onset to the onset of evoked muscle potential, was modulated after SCI. Before injury, the latency of the response in the triceps was significantly shorter on the ipsilesional side if the ipsilesional sciatic was stimulated. Similarly, the response was shorter on the contralesional triceps when the contralesional sciatic was stimulated (Table 2). After hemicontusion, the MIER latency on the contralesional side was delayed compared to the ipsilesional side whether the stimulation was evoked from either the ipsilesional or contralesional sciatic (Table 2). In addition, MIER amplitude was significantly larger in the ipsilesional triceps when compared to contralesional triceps at 1 and 6 weeks and also was larger than baseline (Figure 3B). We confirmed that signals could not be transmitted through the injury by attempting to record EMGs from the masseter muscle (*data not shown*). The MT for activation of the ipsilesional and contralesional triceps was ~38% of the stimulator output before injury. Six weeks after injury, the threshold to evoke a response in the contralesional triceps was increased to 48% when the ipsilesional sciatic was stimulated and to 43% when the contralesional sciatic was stimulated. The MT for the ipsilesional triceps was not significantly changed after SCI (Table 2).

**Table 2:** **Magnetic inter-enlargement responses and motor-evoked potentials latency and motor threshold 1 week and 6 weeks after SCI**.

			**Baseline**		**Hemicontusion**			
					**Week 1**		**Week 6**	
			**Contra**	**Ipsi**	**Contra**	**Ipsi**	**Contra**	**Ipsi**
**Stimulation**	**MIER contralesional sciatic stimulation**	**Latency (ms)**					
		Mean	5.83	6.17^*^	6.12	5.53^*^^§^	6.04	5.57^*^^§^
		SEM	0.09	0.12	0.20	0.15	0.19	0.13
		**Motor threshold**					
		Mean	38%	38%	42%^*^	33%	43%^*^	35%
		SEM	2%	2%	4%	2%	2%	2%
	**MIER ipsilesional sciatic stimulation**	**Latency (ms)**					
		Mean	6.50	6.24^*^	5.82	5.53^*^^§^	6.07	5.61^*^^§^
		SEM	0.14	0.13	0.16	0.15	0.15	0.14
		**Motor threshold**					
		Mean	37%	38%	42%^*^^§^	37%	48%^*^^§^	37%
		SEM	3%	2%	3%	4%	3%	2%
	**MMEP**	**Latency (ms)**					
		Mean	6.04	6.04	6.27	**6.73^§^**	6.09	**7.23^§^**
		SEM	0.06	0.07	0.10	0.38	0.11	0.30
		**Motor threshold**					
		Mean	31%	31%	31%	35%	31%	43%^*^^§^
		SEM	1%	1%	2%	4%	2%	4%

Contra, contralesional; Ipsi, ipsilesional. NA indicates no response.

§ indicates a significant difference vs. baseline

* a significant difference vs. contralesional.

The ascending signal between the lumbar and cervical enlargement appeared to be enhanced after injury on the side of the lesion. To rule out any unwanted effect of magnetic stimulation, we further investigated transmission in this pathway using electrical stimulation in a subset of animals. Cuff electrodes were implanted bilaterally on the sciatic nerve for stimulation and recordings were performed as described using needle electrodes. Magnetic and electrical stimulation yielded similar results, i.e., the amplitude of the response was much larger in the ipsilesional as compared to the contralesional triceps no matter if the contralesional or ipsilesional sciatic was stimulated (Figure 3C). Before injury, the MIER amplitude response in either triceps was comparable. The amplitude of the MIER in the ipsilesional triceps was increased after SCI by ~2.5 fold whether we used magnetic or electrical stimulation (Figure 3B). The amplitude of MIER did not correlate with recovery of locomotion.

### Transmission in descending pathways is impaired on the ipsilesional side after incomplete SCI

**Figure 4:**
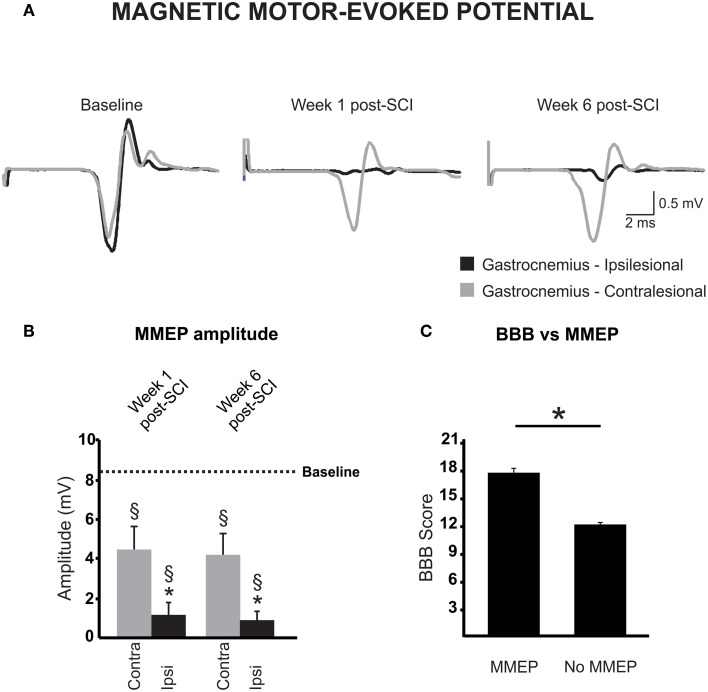
**Magnetic motor-evoked potential (MMEP) partly recovers after a cervical hemicontusion injury. (A)** MMEPs recorded from the contralesional (gray) and ipsilesional (black) gastrocnemius (GS) muscles disappear on the ipsilesional side after SCI and partly recovers over time. MMEPs are delayed and of smaller amplitude **(B)** in the ipsilesional GS (black) as compared to baseline (dotted line) or the contralesional GS at matched times (gray). **(C)** Animals with no MMEP in the ipsilesional GS exhibited a lower extent of locomotor recovery in the open field.

We assessed conductivity and connectivity of descending spared fibers after hemicontusion injury over 6 weeks post injury in awake animals using transcranial magnetic stimulation to elicit MMEPs in the right and left gastrocnemius (GS) muscles. Figure 4A illustrates averaged traces recorded with a stimulus delivered at 60% of the maximal output of the stimulator for both hemicontused animals. We found no difference in response latency in either GS before injury or between the contralesional GS at 1 or 6 weeks vs. baseline (Table 2). However, MMEP amplitude was significantly decreased in the contralesional GS at 1 week post-SCI and 6 weeks post-SCI as compared to baseline (Figure 4B). Also, the response in the ipsilesional GS remained delayed (~1.2 ms) and of significantly smaller amplitude compared to both baseline and contralesional GS at matched times at least up to 6 weeks post-SCI. To validate these results, we confirmed that animals with C5 lateral hemisection and complete disruption of descending pathways on the ipsilesional side exhibited no MMEP in the ipsilesional GS (*data not shown*).

The MT, i.e., the lowest stimulation intensity that evoked a response at least 50% of the time, was also measured at baseline and after SCI. As reported by others, all animals exhibited responses at 40% stimulation level, a majority at 30%, and a few at 20% (Linden et al., 1999). Six weeks after injury, 10 animals failed to display MMEPs in the ipsilesional GS even at 100% of the stimulator output. For those animals who presented MMEPs, they arose with stimulus intensity between 50 and 60%. Six weeks after hemicontusion injury, activation threshold was significantly increased by 12% in the ipsilesional GS but was not changed at 1 week post-SCI (Table 2). Importantly, the amplitude of the ipsilesional MMEP response positively correlated to the ipsilesional hindlimb BBB score (ρ = 0.462; *p* = 0.03). Animals with MMEPs in the ipsilesional GS exhibited better recovery of locomotion in the open field compared to those with no ipsilesional MMEP (Figure 4C).

### The frequency-dependent depression of the H-reflex after unilateral SCI is impaired in both forelimbs but not in the hindlimbs

**Figure 5:**
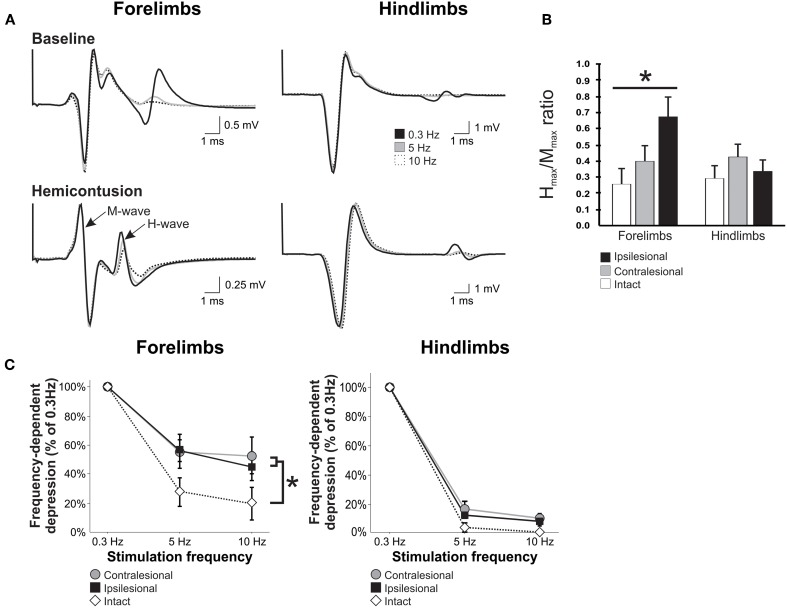
**Unilateral cervical SCI affects H-reflex modulation bilaterally in the forelimbs, but not in the hindlimbs.** H-reflex traces were evoked by the stimulation of the tibial or ulnar nerve and recorded from the interosseus muscle of ipsilesional and contralesional hindlimb and forelimb. **(A)** Representative average of H-reflex recordings following a train of stimulation at 0.3 Hz (black), 5 Hz (gray), and 10 Hz (white) in normal and SCI animals. There was a decrease in the average amplitude of the H-reflex with increasing stimulus frequency in the hindlimbs and forelimbs of a normal animal and also in the hindlimbs of an SCI animal. However, this decrease was modest in the impaired forelimb of the hemicontused animal. **(B)** H_max_/M_max_ ratio is increased in the ipsilesional forelimb after SCI but not in the contralesional forelimb or the hindlimbs. **(C)** Overall, the frequency-dependent depression of the H-reflex displayed in the hindlimbs of SCI animals is similar to normal animals 8 weeks after injury. However, the depression is impaired in both forelimbs as compared to normal.

Stimulation of the tibial nerve evoked two consecutive muscle responses, the M and the H waves (Figure 5A). H-reflex gain and threshold were determined from recruitment curves generated with stimuli of increasing amplitude. No significant difference was observed in the electrical threshold to evoke a motor response (M-wave) after injury (*data not shown*). Similarly, the threshold for H-reflex initiation (H-wave) was unchanged in the hindlimbs but it was increased in the ipsilesional side (2.62 ± 1.19MT) as compared to the contralesional side (1.16 ± 0.25MT) or normal forelimbs (1.16 ± 0.14MT). Eight weeks after injury, there was an increase in H_max_/M_max_ ratio in the ipsilesional forelimb (0.67 ± 0.13) but not in the contralesional forelimb (0.40 ± 0.09) as compared to non injured animals (0.26 ± 0.09, Figures 5B,C). The same ratio was not significantly different in the hindlimbs 8 weeks SCI.

Averaged H-reflex recordings evoked by train of stimulation at 0.3 Hz (black), 5 Hz (gray) and 10 Hz (dotted) in the ispi- and contralesional forelimb and hindlimbs from hemicontused animals are illustrated in Figure 5A. These recordings demonstrate that increasing the frequency of stimulation leads to a substantial decrease in H-reflex amplitude in the forelimbs and hindlimbs of normal animals. As a group, hemicontused animals displayed a significant modulation of the H-reflex in the hindlimbs with increasing stimulus frequency with depression values similar to normal animals. The greater the depression, the lower the value is on the *y axis.* Although FDD of the H-reflex was not impaired in the hindlimbs, it was significantly impaired in both the ipsilesional and contralesional forelimbs at 5 Hz and 10 Hz as compared to the normal group. A Two-Way ANOVA revealed statistically significant differences across stimulation frequency, across experimental groups, and an interaction between frequency and groups. *Post-hoc* comparisons showed that 5 Hz and 10 Hz depression values were different than 0.3 Hz in all limbs and groups and that hemicontused forelimb values were different than normal animals both at 5 Hz and 10 Hz. At 10 Hz, there was 34% less depression in the contralesional and 26% in the ipsilesional forelimb. These results suggest that the excitability of cervical motor pools, that are located several spinal segments below the lesion, is affected in a bilateral manner.

### Contributions of spared white matter to MMEP and MIER responses

**Figure 6:**
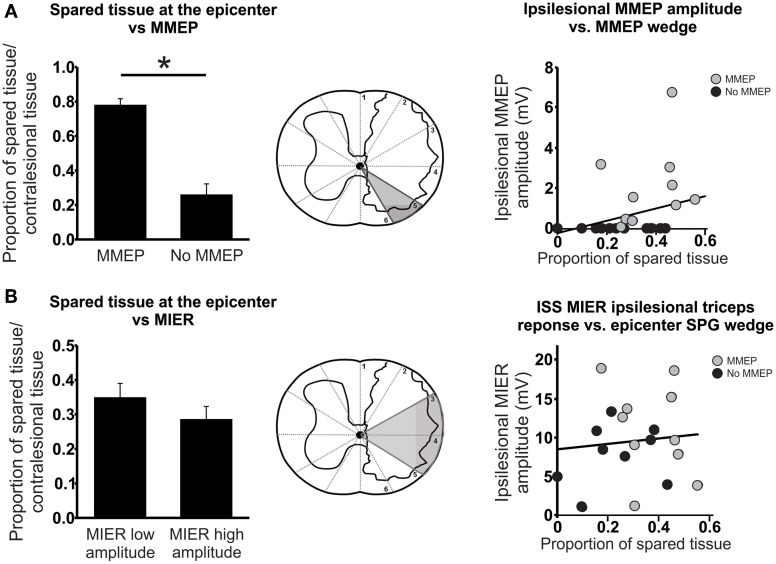
**Anatomical evaluation of tissue sparing related to the MMEP or the MIER responses. (A)** Magnetic motor evoked potentials (MMEP) are transmitted in the ventrolateral white matter of the spinal cord. Animals that failed to exhibit ipsilesional MMEPs had significantly less spared tissue within this ventrolateral portion of the spinal cord at the lesion epicenter. Correlational analysis revealed a significant relationship between the amplitude of the MMEP response and the proportion of tissue sparing in the ventrolateral white matter responsible for MMEP transmission (gray wedge of schematic). **(B)** Magnetic Inter-Enlargement Responses (MIER) travel through the lateral white matter of the spinal cord. The amount of tissue sparing within these regions at the lesion epicenter did not affect the amplitude of the MIER. A scatter plot and correlational analysis failed to show a relationship between the amplitude of the ipsilesional MIER and the proportion of tissue sparing in the lateral white matter (gray wedge of schematic).

A previous report by Loy et al. (2002) determined that the ventrolateral white matter of the spinal cord is necessary to elicit an MMEP response in the GS muscle. In order to limit the analysis of white matter sparing to the ventrolateral spinal cord, we determined the amount of sparing in the gray “ventrolateral” wedge depicted in Figure 6A. This analysis of the lesion site revealed that the presence of an ipsilesional MMEP was directly related to the amount of sparing in the ventrolateral white matter of the spinal cord (Figure 6A). Regression and correlational analysis using non-parametric Spearman's rank correlation test showed that animals with no ipsilesional MMEP had significantly less spared tissue within the ventrolateral white matter than those demonstrating bilateral MMEPs (Figure 6A; Spearman's ρ = 0.544; *p* < 0.009; *r*^2^ = 0.35).

We examined the dorsolateral and lateral spinal cord responsible for eliciting a MIER response in a similar manner (Beaumont et al., 2006; Figure 6B). By partitioning the animals into two groups with low or high amplitude responses and comparing the proportion of spared tissue in the dorsolateral and lateral wedge (Figure 6B) we saw no effect of sparing on the amplitude of the MIER response (Figure 6B). Likewise, regression and correlational analysis using Spearman's rank test showed no significant relationship between MIER amplitude and the amount of tissue sparing at the epicenter, indicating that the fibers responsible for transmitting the MIER signal are located in the lateral-most edge of the dorsolateral white matter (Figure 6B; Spearman's ρ = 0.0.007; *p* < 0.097; *r*^2^ = 0.009).

**Figure 7:**
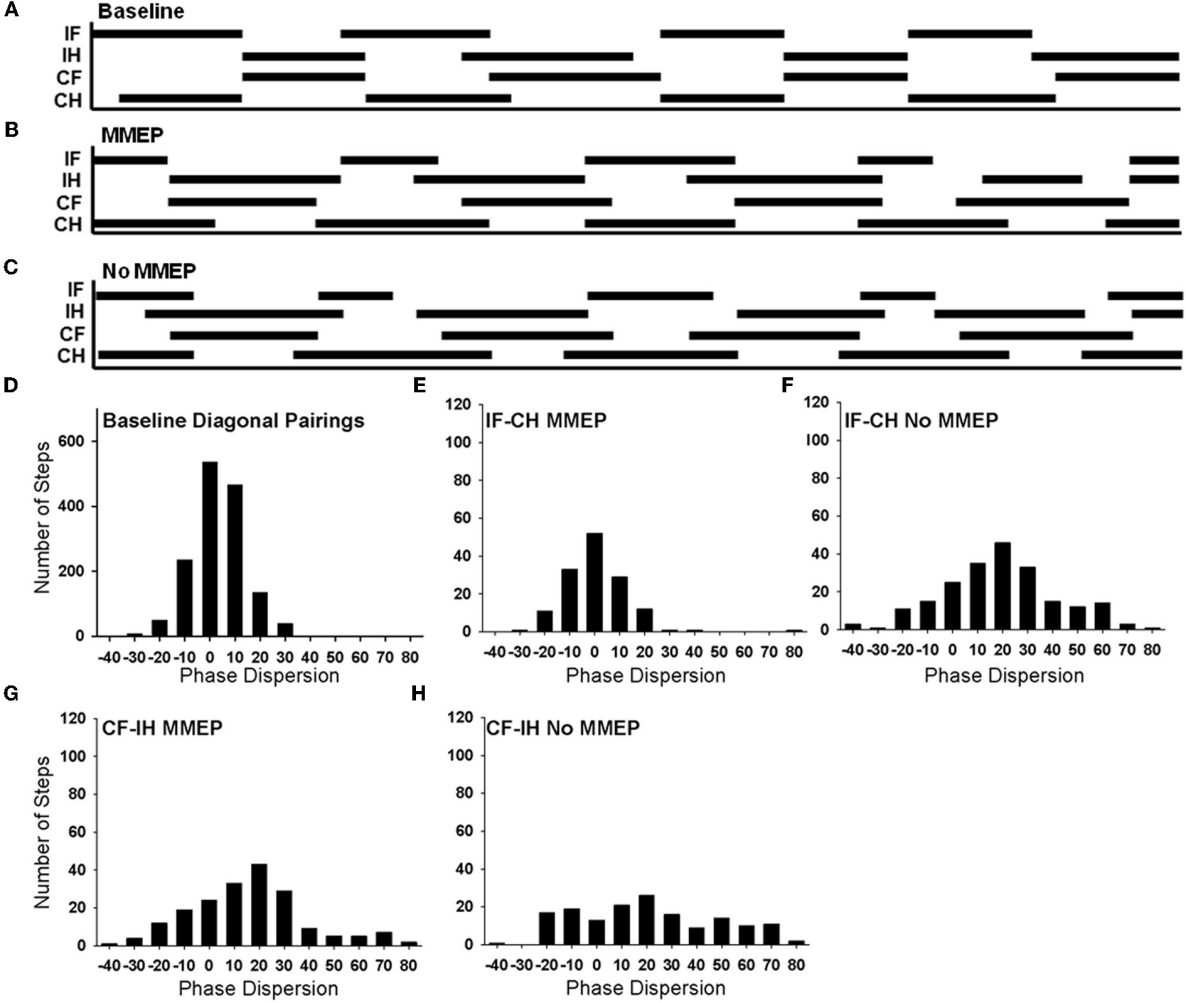
**Analysis of forelimb–hindlimb coordination in animals with and without ipsilesional MMEPs 6 weeks after SCI.** Footfall diagrams during CatWalk locomotion for normal **(A)**, SCI animals with **(B)**, and without **(C)** ipsilesional MMEP responses allow the comparison of movements of all four limbs (IF, ipsilesional forelimb; IH, ipsilesional hindlimb; CF, contralesional forelimb; CH, contralesional hindlimb). The stance phase of each limb is indicated by black bars and the swing phase is indicated by the open spaces. Overlap of the solid lines for the MMEP and No MMEP groups indicate times when the limbs are moving synchronously. Comparison of the correspondence of stance and swing phase reveals a distribution pattern or phase dispersion for normal animals that centers at 0%, indicating that the initial contact of both diagonal limb pairings are tightly coupled **(D)**. **(E)** Animals with ipsilesional MMEP recovered a synchronous coordinative relationship that was similar to baseline values for the ipsilesional FL-contralesional HL limb pairing, yet for the other diagonal limb pairing (contralesional FL-ipsilesional HL; **(G)**, animals with ipsilesional MMEPs adopted a more lax strategy to coordinate the forelimbs and the hindlimbs as seen by a positive shift of the mean phase dispersion compared to baseline values and greater variability in the histogram. Animals with no ipsilesional MMEP demonstrated a lower degree of recovery of FL–HL coordination **(F,H)**. For the ipsilesional FL-contralesional HL limb pairing for this group of animals showed a positive shift in the mean phase dispersion compared to baseline values. The variability covered a broad range of –20% to +60%. There was no evidence of a predominant coordinative pattern for the contralesional FL-ipsilesional HL in animals with no ipsilesional MMEP **(H)**. A significant shift of mean phase dispersion (~20%) and the values of phase dispersion were evenly distributed across the range –20% to +70%.

Interlimb coordination is a complex skill based on the ability to consistently implement a predominant, rhythmic pattern with little variability. Animals with no MMEP response in the ipsilesional GS muscle exhibited deficits in forelimb–hindlimb (FL–HL) coordination. In the open field, all animals with bilateral MMEPs were able to execute FL–HL coordination more than 95% of the time (BBB = 14), while animals with no ipsilesional MMEP exhibited FL–HL coordination <50% of the time. In order to examine FL–HL coordination in greater detail, we created footfall diagrams of CatWalk passes to allow us to determine how all four limbs move relative to each other (Figures 7A–C). Solid lines represent the time a limb is in stance and open spaces represent time when the limb is in the swing phase of locomotion. In normal animals, the ipsilesional forelimb and contralesional hindlimb move through stance and swing phase in concert (Figure 7A). The footfall pattern changes with SCI (Figures 7B,C). The ability to abruptly transition from one diagonal pairing to the other is not retained with injury, as depicted by instances of three or more limbs in stance at one time. Furthermore, quantification of these changes in the transition between diagonal pairings by determining the mean phase dispersion 6 weeks after SCI revealed that animals without ipsilesional MMEPs exhibited a significant deficit in their ability to implement a single coordinative strategy during bouts of runway locomotion. The deficits in coordination were associated with FL–HL limb pairing rather than those across the shoulder or pelvic girdles.

Figure 7C shows a histogram of the phase dispersion for both diagonal limb pairings in normal animals. For each diagonal pairing (IF-CH or CF-IH) the FL and HL are in phase with each other (i.e., the FL and HL initiate initial contact at the same time), with a mean phase dispersion at 4.36%. Normally, phase dispersion values for diagonal limb pairings are tightly distributed between –20% and 20%, and the standard deviation is small (7.65 + 0.62%) (Figure 7D). Regardless of the presence of ipsilesional MMEP, SCI animals demonstrated better synchrony of the IF-CL compared to the CF-IH pairing. Animals with bilateral MMEPs demonstrated tight synchrony between the IF and CH (Figure 7E, mean phase dispersion: 0.72 + 1.26%) that was not different than baseline. Animals with no ipsilesional MMEP produced a positive shift in the coincidence (mean phase dispersion: 18.64 + 7.26%, Figure 7F) suggesting that the coordination for the IF-CH is approximately 20% out of phase compared to normal. Standard deviation of the phase dispersion remained small, indicating that animals with MMEPs were able to consistently implement a single coordinative strategy for this limb pairing (Baseline: 7.26 + 2.5%; MMEP: 8.31 + 0.1.3%) with the coincidence ranging from −20% to +20%. Animals with no ipsilesional MMEP exhibited significantly greater variability in phase dispersion compared to normal and animals with MMEPs (No MMEP: 14.38 + 1.43%) that extended over a range of –20% to +60%.

Deficits in coordination were most visible in the CF-IH pairing. While the distribution of the phase dispersion appeared similar to normal, animals with MMEP exhibited a positive shift in mean phase dispersion and increased variability compared to normal (Figure 7G; mean phase dispersion: baseline: 4.36%, MMEP:16.38 + 6.21%; standard deviation: baseline 7.65 + 0.62%, MMEP: 17.12 + 1.34%). Animals with no ipsilesional MMEP were unable to execute synchronous placement of the CF and IH during runway locomotion (Figure 7H). Their mean phase dispersion was shifted 19.29 + 15.22% and the variability of this limb pairing was 26.5 + 2.64%. For the CF-IH relationship, phase dispersion values were evenly distributed across the range −20% to +70%. All animals, regardless of MMEP response, showed an increase in the incidence of mismatched limb pairings compared to normal.

## Discussion

It is now recognized that long distance regeneration might not be necessary to contribute to functional recovery after SCI; a short distance regeneration and/or formation of new relays appears to be sufficient (Jordan and Schmidt, 2002; Bareyre et al., 2004; Courtine et al., 2008). Long descending propriospinal neurons have been shown to be involved in new circuits that are reorganized after SCI and act as a relay between the cortex and their spinal target of origin (Bareyre et al., 2004). Propriospinal neurons thus are well suited to accomplish this role and the localization of their axons in the lateral and ventral white matter make them easily identified targets for therapeutic strategies after SCI (Conta and Stelzner, 2004). The results presented here extend these findings by characterizing plasticity occurring in ascending propriospinal networks after SCI. We show enhanced transmission in ascending propriospinal pathways and a concomitant increase in H_max_/M_max_ ratio in the ipsilesional triceps. Surprisingly, while MMEPs correlated with tissue sparing and locomotor recovery, MIER did not show any relationship with these outcomes and did not predict better interlimb coordination.

### Increased transmission in ascending long propriospinal fibers

Long ascending propriospinal neurons that originate in the lumbar enlargement and terminate in the cervical enlargement have been anatomically identified, shown to ascend in the ipsilateral VLF and to send collaterals to the contralateral side of the spinal cord (Giovanelli and Kuypers, 1969; Reed et al., 2006, 2009). Also, the stimulation of the peroneal nerve in humans can elicit responses in arm muscles (Zehr et al., 2001) that are modulated by leg movements during walking in a phase-dependent manner (Zehr and Haridas, 2003; Zehr et al., 2007). We used a recently described electrophysiological measure of the circuitry between the lumbar and cervical enlargements (Beaumont et al., 2006) to address the possible contribution of this pathway to functional recovery after SCI. We confirmed that signals could not be transmitted through the injury by attempting to record EMGs from the masseter muscle which is known to receive inputs from group II afferents of the sciatic nerve (Deriu et al., 2001, see also Beaumont et al., 2006). While we recorded no signals in the masseter muscle, we found that transmission between the lumbar and cervical enlargement was increased ipsilaterally after a hemicontusion injury, with no effect on the contralateral side. Volume-conducted transmission from the site of stimulation or direct stimulation of cervical muscles that would then conduct the stimulus caudally to the recording site is unlikely since the response was absent in some animals or in any of the masseter muscles we recorded from. In addition, a similar artifact was never observed with the MMEP which was not enhanced in any group or experimental condition.

The mean latencies of the MIER at baseline ranged between 5.83 ms to 6.5 ms (see Table 2). With a distance between the sciatic at the iliac crest and the cervical enlargement of ~105 mm, the conduction velocity including synaptic delay would be ~16−18 m/s similar to what has previously been reported (Beaumont et al., 2006). Assuming a conduction velocity of 30 m/s (Carp et al., 2003), the synaptic delay would range from 2.33 to 3 ms, and the pathway potentially involve 3−5 synapses. Before injury, we observed that the response latency was significantly longer in the triceps contralateral to the hip stimulated. Whether this difference reflects an extra synapse on a commissural interneuron or added distance to travel across the cord remains to be determined. After injury, the latency was shorter on the ipsilesional side no matter which sciatic (contra or ipsi) was stimulated. Conduction velocity has been shown to be influenced by firing/depolarization threshold (Carp et al., 2001, 2003) which, itself, is modulated by activity and neuromodulators, two factors that are considerably affected by SCI. The inverse relationship between firing threshold and conduction velocity, suggest that the decrease in MIER latency that we observe after SCI on the ipsilesional side could rely on the increased excitability of the forelimbs motoneuronal pools as evidenced by the increased H_max_/M_max_ ratio. More experiments would be necessary to address this question.

Ascending propriospinal neurons are involved in the coupling of lumbar and cervical CPGs and also interlimb coordination (Miller et al., 1973a,b), allowing the lumbar circuitry to drive or entrain the cervical enlargement (Juvin et al., 2005) and imposing a left-right alternation on the cervical networks. Behaviorally, we showed that our animals had not fully recovered appropriate interlimb coordination. Proper activation of the triceps brachii, a forelimb extensor, is crucial during locomotion and postural tasks which require coordination between the hindlimbs and forelimbs (Jordan and Schmidt, 2002). We did not observe a significant relationship between white matter sparing in the LF/VLF, BBB scores, and the amplitude of the MIER as previously reported in a thoracic contusion animal model (Beaumont et al., 2006). This is not surprising since our cervical unilateral injury was located rostral the triceps motor pool and did not directly affect the fibers that transmits the MIER whereas a thoracic contusion injury directly damage the MIER pathway. The increased transmission between the sciatic nerve and triceps brachii we observed with the MIER could be due to facilitation and/or disinhibition, both of which have been shown to occur after SCI. Disruption of supraspinal inhibitory pathways was confirmed by the analysis of the lesion site. Our experimental design did not allow us to determine if facilitation due to sprouting or increased synaptic strength also contributed to enhance the MIER in the ipsilesional triceps, although an increase in H_max_/M_max_ suggests an increase in excitability of the ipsilesional motoneuron pool. Further study of how enhancement of intersegmental transmission to ipsilesional forelimb extensor muscles contributed to spontaneous functional recovery would require a detailed EMG and kinematics analysis.

### Decreased transmission in descending motor pathways

Use of MMEPs as a functional measurement of motor pathway damage after SCI (Magnuson et al., 1999) depends on the activation of subcortical structures (Kamida et al., 1998) and the transmission of generated signals through axons that travel in the VLF of the spinal cord (Linden et al., 1999; Loy et al., 2002). It has been suggested that transcranial magnetic stimulation excites fast pathways (~50 m/s) which reflect rodent motor function more accurately than the dorsal corticospinal tract that is activated by electrical motor-evoked potentials (MEP, Kamida et al., 1998; Luft et al., 2001) but not by MMEPs (Ryder et al., 1991). Therefore, MMEPs have great advantages over MEPs to assess motor recovery after SCI since the corticospinal tract is not crucially involved in locomotion whereas the ventral spinal cord is the main carrier of locomotion and postural control (Basso et al., 1996).

There is still debate as to whether the VLF carries descending fibers that are necessary (Steeves and Jordan, 1980; Noga et al., 1991) or not for the locomotor pattern to be expressed (Vilensky et al., 1992; Brustein and Rossignol, 1998) because of the redundancy of the pathway in the ventral column (Loy et al., 2002). Here, all our animals spontaneously recovered some degree of coordinated locomotion. The partial preservation of the ventral white matter tracts may account for the spontaneous locomotor recovery we observed. Sparing of 10−15% of ventral/lateral white matter is sufficient for locomotor movements after SCI (Schucht et al., 2002), while complete lesions of ventral white matter prevent the recovery of stepping. This suggests that sparing of at least part of the reticulospinal tract is necessary to initiate stepping and the recovery of rat locomotion (Loy et al., 2002). In our experiment, animals that did not present MMEPs had lower BBB scores than animals which recovered MMEP on the ipsilesional side. Analyzing specific regions of the ventral and lateral funiculi that contain the reticulospinal fiber tract that carries MMEP signals was a better predictor of locomotor recovery than overall white matter sparing at the lesion site (Schucht et al., 2002). MMEP latency remained delayed in the ipsilesional side 6 weeks after injury. We feel that this delay could arise from demyelination of spared fibers, formation of a new relay with propriospinal interneurons or unmasking of a previously “silent” polysynaptic pathway. It is likely that the return of MMEP signal over time represents the return of function in spared fibers rather than reorganization through the contralateral side since signal remained absent in hemisected animals even 6 weeks after the injury and the amplitude of the MMEP in the contralesional GS was ~50% of pre-injury values. This decrease was not the result of damage to the contralesional cervical spinal cord since the white matter on the contralesional side appeared contiguous, did not have vacuoles and did not show anatomical evidence of demyelination.

Interlimb coordination during locomotion is a multifaceted skill that requires the ability to sustain a predominant rhythmic pattern with limited variability during locomotion that results in one-to-one limb movements. Interlimb coordination has been most often studied in laboratory animals using the BBB Locomotor Rating Scale. The evaluation of interlimb coordination using the BBB relies on visual assessment of a one-to-one step ratio between the FLs and HLs, and due to the live nature of BBB testing, the consistency of the patterned movements cannot be accurately determined (Kloos et al., 2005). Phase dispersion is a highly sensitive method to measure interlimb coordination. It quantifies three components of interlimb coordination: the pattern, consistency, and step ratio. Kloos et al. (2005) used these three values to identify two degrees of coordination in mild SCI that were indistinguishable using the BBB. They termed these two groups a synchronous group where animals consistently and repeatedly displayed a one-to-one coordinative relationship between limb pairs, and modified concordance where animals implemented a more lax coordination strategy. Here, our examination of interlimb coordination using BBB showed significant differences between animals with and without ipsilesional MMEPs, and phase dispersion was able to further identify differences in the coordination of diagonal pairings after unilateral cervical SCI. That phase dispersion is a precise indicator of the pattern and underlying variability of limb placement during locomotion suggests that it may be a predictor of spared ascending and descending fibers necessary to mediate FL–HL coordination.

### Excitability of local cervical spinal networks

The H-reflex is a measure of both peripheral sensory and motor nerve conductivity as well as segmental integrity of function (Thompson et al., 1998, 2001). After SCI, physiological and biomolecular changes such as ischemia, inflammation, and modification of neurotransmitter expression at the epicenter and beyond can affect the M- and H-wave of the H-reflex. Injury leads to increased reflex excitability (Thompson et al., 1992, 1998, 2001; Hiersemenzel et al., 2000), disruption of serotonergic descending pathways (Schmidt and Jordan, 2000) and enhanced monosynaptic transmission of group Ia fibers (Cope et al., 1988). The functional and anatomical changes that occurred in the cervical spinal cord are reflected by the increase in the H_max_/M_max_ ratio in the ipsilesional forelimb in our experiments.

Although a change in H_max_/M_max_ ratio is typically thought to reflect altered motoneuron excitability, this one-dimensional assumption is widely contested in the literature as many other factors can contribute to the modulation of the amplitude of the H- and/or M-wave. Amplitude of the H- and M-wave varies significantly with stimulus intensity and a variety of factors contribute to set the input-output gain across a motoneuronal pool (reviewed in Hultborn et al., 2004). Among those, the slope of the ascending part of the recruitment curve can provide information about the input–output relationship (Mazzocchio et al., 2001). The reflex gain can also be estimated as the slope between motoneuron activation (measured as background activity) and H-reflex amplitude. Although it is essential to determine background EMG activity for studies carried out during movement, its effect is minimal under anesthesia and our baseline recordings were silent. We found no difference in steepness of the slopes of the H-reflex or M-wave recruitment curve for stimulation on either the contra- or ipsilesional side (*data not shown*). Conversion of muscle fibers toward faster phenotypes has been shown to occur after SCI and/or unloading and could have a marked effect on the amplitude of the M-wave (Roy et al., 1991; Talmadge, 2000). However, this increase in FF motor units is transitional after contusion injury, gradually returns to normal level and correlates with weight-bearing recovery (Hutchinson et al., 2001; Roy et al., 2011). Therefore, a switch in muscle unit properties is unlikely to have contributed to changing the H_max_/M_max_ ratio as our animals have recovered weight-bearing in the forepaws by 3 weeks post-SCI and the amplitude of the M-wave was not significantly different between intact animals and the ipsi- or contralesional forepaw 8 weeks after hemicontusion injury. The amplitude of H_max_ depends on pre and post synaptic events and concomitant antidromic activity elicited in motor nerves (Misiaszek, 2003; Knikou, 2008). The advantage of using the H-reflex by stimulating the nerve (vs. stretch reflex) is that it bypasses the muscle spindles and fusimotor activity that could influence the sensitivity of Ia afferents. Also, when reflexes are recorded at rest as it is the case in this study, the influence from supraspinal pathways are anticipated to be minimal and Renshaw cells and Ia/Ib interneurons less active. Although other mechanisms cannot fully be ruled out, the model we used here tended to limit the effect of other confounding factors and we believe the change in H_max_/M_max_ ratio truly represents a change in motor pool activity in this context.

Our injury altered local cervical reflex excitability in the ipsilesional forelimb only, but decreased FDD bilaterally suggesting that the injury induced changes in motoneuronal properties and motor pool excitability only on the ipsilesional side. The contralesional cervical neurons did not seem to be affected by the injury in the same manner with a H_max_/M_max_ ratio similar to the normal group. The ipsilesional increase in H_max_/M_max_ ratio may be due to increased sprouting of Ia afferents onto motoneuronal dendrites with reduced supraspinal inputs; similar openings onto the dendritic arbor would not be observed contralesionally as the supraspinal inputs are for the most part preserved on that side. A possible explanation for the contralesional decrease in FDD would be the lack of presynaptic inhibition from ipsilesional collaterals onto Ia afferents of the contralesional triceps. Changes in the H-reflex pathway after SCI suggest both spinal reorganization and an associated decrease in presynaptic inhibition (Schindler-Ivens and Shields, 2000) with some of the effects being unilateral and others bilateral. Of supraspinal neurons, only reticulospinal neurons appear to contribute to presynaptic inhibition of group Ia afferents in any major way; the axons of these neurons run in the medial longitudinal fascicle and the ventral quadrant of the spinal cord (Carpenter et al., 1963, 1966; Reed et al., 2008) which are greatly compromised in our model.

## Summary

Two months after a unilateral contusive injury to the C5 spinal cord, animals demonstrated a persistent disruption of signal transmission in ascending and descending pathways despite partial functional recovery. Our results indicate that complex plasticity occurs, leading to differential changes in both the ipsi- and contralesional spinal networks even though the lesion was unilateral. Animals that did not recover MMEPs on the ipsilesional side had significantly less spared tissue and presented inconsistent interlimb coordination as compared to those who recovered MMEPs. The lack of relationship of the MIER to functional outcomes prevents us to establish a clear role of ascending propriospinal pathways in either partial recovery or lasting deficits we observed. Nonetheless, that this pathway is intact and excitable with stimulation suggests that with the right type of stimulation (exercise, epidural stimulation) it may represent a potential avenue to further drive and improve locomotor recovery.

### Conflict of interest statement

The authors declare that the research was conducted in the absence of any commercial or financial relationships that could be construed as a potential conflict of interest.
